# Vaginal cytokine profile and microbiota before and after lubricant use compared with condomless vaginal sex: a preliminary observational study

**DOI:** 10.1186/s12879-021-06512-x

**Published:** 2021-09-18

**Authors:** Susan Tuddenham, Christina A. Stennett, Richard A. Cone, Jacques Ravel, Andrew N. Macintyre, Khalil G. Ghanem, Xin He, Rebecca M. Brotman

**Affiliations:** 1 Department of Medicine, Johns Hopkins University School of Medicine, Baltimore, MD USA; 2 Institute for Genome Sciences, University of Maryland School of Medicine, Baltimore, MD USA; 3 Department of Biophysics, Johns Hopkins University, Baltimore, MD USA; 4 Department of Medicine, Duke Human Vaccine Institute, Duke University School of Medicine, Durham, NC USA; 5 School of Public Health, University of Maryland College Park, College Park, MD USA

**Keywords:** Vaginal lubricants, Immunology, Bacterial vaginosis, Sexually transmitted infections

## Abstract

**Background:**

Limited data suggest that personal lubricants may damage the vaginal mucosal epithelium, alter the vaginal microbiota, and increase inflammation. We compared vaginal cytokine profiles and microbiota before and after vaginal lubricant use and condomless vaginal sex.

**Methods:**

Reproductive-age women were recruited to a 10-week observational cohort study and were asked to self-collect vaginal samples and behavioral diaries daily. This nested case–control analysis utilized samples collected before and after self-reported condomless sexual activity with lubricants (22 case participants) and without lubricants (22 control participants). Controls were matched to cases on race/ethnicity. Microbiota composition was characterized by sequencing amplicons of the 16S rRNA gene V3–V4 regions. Cytokine concentrations were quantified using a magnetic bead 41-plex panel assay and read using a Bio-Plex 200 array reader. Wilcoxon signed-rank tests were used to assess baseline differences in vaginal cytokines between cases and controls as well as differences pre- and post-exposure. Linear mixed effects models were used to examine differences in relative post-to-pre change in each individual cytokine between matched cases and controls. Similar analyses were conducted for the microbiota data.

**Results:**

Mean age was 29.8 years (SD 6.8), and 63.6% were African American. There were few statistically significant changes in cytokines or microbiota before and after exposure in cases or controls. In mixed-effects modeling, the mean relative post-to-pre change of cytokines was higher in cases vs. controls for macrophage derived chemokine (MDC) (p = 0.03). The microbiota data revealed no significant changes when measured by similarity scores, diversity indexes and descriptive community state types (CST) transition analyses. However, post sexual activity, the mean relative abundance of *L. crispatus* decreased for those who used lubricants (particularly those who were *L. iners*-dominated prior to exposure)**.**

**Conclusions:**

Although there were overall few differences in the vaginal microbiota and cytokine profiles of lubricant users and controls before and after condomless vaginal sex, there was a trend toward decreases in relative abundance of *L. crispatus* following use of lubricant. Future larger studies that take into account osmolarity and composition of lubricants may provide additional insights.

**Supplementary Information:**

The online version contains supplementary material available at 10.1186/s12879-021-06512-x.

## Background

Over 60% of American women report use of personal lubricants during intimate sexual activities [1]. Vaginal lubricants are also often administered to improve comfort during clinical gynecological exams. As most of these products are classified by the Food and Drug Administration (FDA) as cosmetics, human safety data is not required prior to commercialization. The FDA classifies lubricants for clinical use as class II medical devices, a category that may pose moderate risk to high to patients [2]. Prior work has linked vaginal products containing nonoxynol-9 (a spermicide) to increased inflammation and risk of HIV acquisition in women [3]. Of concern, non-spermicide containing lubricants have been linked to enhanced vaginal susceptibility to sexually transmitted infections (STIs) in animal models [4] and to an increased risk of rectal STIs in men who have sex with men [5].

The mechanisms for this enhanced risk with non-spermicide containing lubricants are unclear, but may relate to mucosal epithelial damage and increased inflammation as well as to changes in local bacterial populations, because lubricants are often formulated with anti-microbial preservatives such as chlorhexidine [6, 7]. Studies have suggested that some lubricants may inhibit the growth of *Lactobacillus* species, an essential component of an optimal vaginal microbiota, thus promoting vaginal dysbiosis [6, 8–11]. This may be important as lack of vaginal lactobacilli, in particular lack of *Lactobacillus crispatus* dominance, has been shown to be associated with significant increase in susceptibility to STIs [12] and HIV acquisition [13]. Additionally, many lubricants are formulated with high concentrations of substances such as glycerol or propylene glycol [14]. These concentrated humectants make them feel comfortably warm when applied to the skin, but also make them markedly hypertonic to mucosal epithelia [6, 14]. Many commercially available lubricants have osmolalities above the World Health Organization (WHO) recommended limit of 1200 mOsm/kg [15, 16]. Multiple studies have shown that hypertonic lubricants may be toxic to mucosal epithelial cells, inducing significant epithelial cell shedding in in-vitro and animal models as well as in humans [6, 7, 17, 18]. For example, one study showed that application of a lubricant with an elevated osmolality caused shedding of large sheets of epithelial cells from rectal tissue [7]. An in vitro study using a three dimensional model of the human vaginal epithelium demonstrated that high osmolality lubricants markedly reduced epithelial barrier properties and induced damage to tissue structures [19].

Elevations in levels of specific “pro-inflammatory” vaginal cytokines have been associated with BV, STIs and enhanced acquisition of HIV [13, 20–22]. Several studies have investigated the impact of vaginal microbicide gels or candidate carrier lubricants on cytokines in the human vagina or upper reproductive tract as potential markers of inflammation, irritation and therefore enhanced HIV acquisition [23–25]. However, none have examined the impact of routine use of vaginal lubricants in women who had condomless vaginal intercourse as compared to condomless vaginal intercourse without lubricant use on both vaginal cytokines and microbiota in a methodical fashion. Despite widespread use, the impact of commercially available vaginal lubricants on the vaginal microenvironment, inflammation and immunity is understudied. Based on our current knowledge, we hypothesized that we would observe a larger increase in (1) pro-inflammatory vaginal cytokines and chemokines and (2) strict and facultative anaerobic bacteria in women reporting use of personal vaginal lubricants (purchased over the counter) during condomless vaginal sex (cases) as compared with women engaging in condomless vaginal sex without lubricant use (controls). In order to test this hypothesis, herein, we compared women’s vaginal cytokine and microbiota profiles before and after condomless vaginal sex with or without vaginal lubricant use.

## Methods

This is a nested case–control study utilizing existing samples from a parent longitudinal study conducted between 2009 and 2010 at the University of Alabama at Birmingham (UAB) [26]. For the parent observational cohort study, HIV-negative, reproductive-age, non-pregnant, cis-gender women were recruited and followed over 10 weeks. All women underwent a pelvic examination and microscopy prior to enrollment to evaluate for symptomatic bacterial vaginosis, vaginal candidiasis, or trichomoniasis. Additionally, an endocervical swab was sent for nucleic acid amplification testing for gonorrhea and chlamydia. If a diagnosis of a STI or symptomatic condition was made at baseline, patients were offered treatment but excluded from the study. Participants were offered the opportunity to return to attempt enrollment again 30 days after treatment was completed. Each day, participants self-collected mid-vaginal swabs and filled out behavioral diaries (yes/no fields). Dry swabs used for cytokine analyses were placed in empty tubes, and swabs used for microbiota analyses were placed in tubes containing Amies transport medium. All swabs were promptly frozen in participants’ home freezers before being returned to the research clinic in weekly batches on frozen ice packs. Once returned, tubes were transferred to long-term storage in −80°C freezers. Samples for cytokine quantification underwent a total of two freeze thaw cycles (one for aliquoting and a final thaw for analysis). All methods were carried out in accordance with relevant guidelines and regulations.

**Fig. 1:**
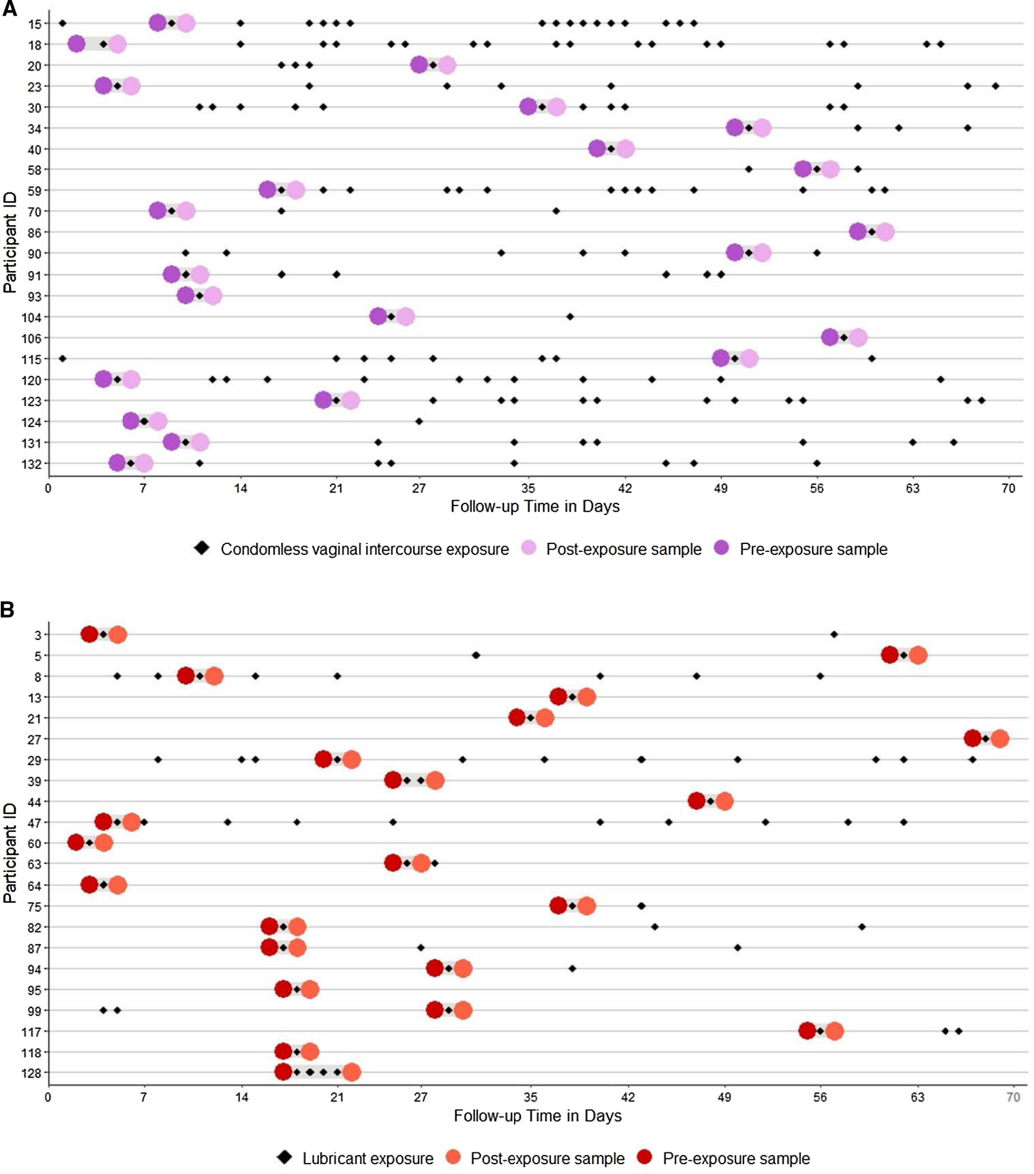
**A** Longitudinal plot for controls (n = 22) including instances of self-reported condomless vaginal sex without lubricant use and pre- and post-exposure samples selected for analysis. **B** Longitudinal plot for cases (n = 22) including instances of self-reported condomless vaginal sex with lubricant use and pre- and post-exposure samples selected for analysis

For this analysis, we first selected vaginal samples collected before and after self-reported lubricant use with condomless vaginal intercourse (cases). For comparison, we then chose vaginal samples from race-matched women collected before and after self-reported condomless vaginal intercourse without report of lubricant use (controls) (Fig. 1A, B). For the majority of participants, an individual exposure day (i.e., one day with reported lubricant use or condomless vaginal sex) was identified, and samples collected on the days immediately before and after this exposure were selected (after confirming these days were exposure-free). For participants with consecutive exposure days, the next appropriate day after exposure ended was selected.

### Cytokine characterization

Prior to analysis, frozen, dry vaginal swabs were eluted into 1 mL of PBS. The eluates were then aliquoted and stored at −80°C until immediately before analysis. Cytokine concentrations in undiluted eluates were quantified in duplicate using a magnetic bead 41-plex panel assay (Millipore HCYTMAG60PMX41BK) performed according to the manufacturer’s recommended protocol and read using a Bio-Plex 200 array reader (Bio-Rad). Data were analyzed using Bio-Plex manager software (Bio-Rad). All samples were assayed in a single batch and manufacturer-provided internal controls were included on each assay plate. The 41 measured cytokines and chemokines included: interferon alpha (INF-α), interferon gamma (INF-γ), interleukin 1 alpha (IL-1α), interleukin 1 beta (IL-1β), interleukin 1 receptor antagonist (IL-1RA), interleukin 2 (IL-2), interleukin 3 (IL-3), interleukin 4 (IL-4), interleukin 5 (IL-5), interleukin 6 (IL-6), interleukin 7 (IL-7), interleukin 8 (IL-8), interleukin 9 (IL-9), interleukin 10 (IL-10), interleukin 12 heterodimer p40 and p35 (IL-12p70), interleukin 12 homodimer p40 (IL-12p40), interleukin 13 (IL-13), interleukin 15 (IL-15), interleukin 17 alpha (IL-17α), tumor necrosis factor alpha (TNF-α), epidermal growth factor (EGF), fibroblast growth factor (FGF), granulocyte colony stimulating factor (GCSF), granulocyte–macrophage colony-stimulating factor (GM-CSF), monocyte chemotactic protein 1 (MCP-1), interferon gamma-induced protein 10 (IP-10), macrophage Inflammatory protein 1 alpha (MIP-1α), macrophage inflammatory protein 1 beta (MIP-1β), tumor necrosis factor beta (TNF-β), vascular endothelial growth factor (VEGF), FMS-like tyrosine kinase 3 ligand (Flt-3L), fractalkine, growth related protein (GRO), monocyte chemotactic protein-3 (MCP-3), macrophage derived chemokine (MDC), platelet derived growth factor AA dimer (PDGFa), platelet derived growth factor AB and BBdimer (PDGFab), soluble CD40 ligand (sCD40L), RANTES (CCL5), eotaxin, and transforming growth factor alpha (TGF-α).

### Vaginal microbiota characterization

Vaginal swabs that had been stored in Amies liquid transport medium at −80°C were selected, thawed, and prepared for genomic DNA extraction. The validated procedures employed have been previously published [26]. In brief, enzymatic and physical lysis of bacterial cells was followed by purification of genomic DNA using a QIAsymphony robotic platform and QIAGEN CellFree 500 kits (QIAGEN, Valencia, CA, USA) according to the manufacturer’s protocol. This procedure provided between 2.5 and 5 μg of high-quality genomic DNA from 300 μl of each sample swab stored in Amies transport. The V3–V4 hypervariable regions of the 16S rRNA genes were targeted from genomic DNA using bacterial primers 338F and 806R and were amplified by a two-step polymerase chain reaction (PCR) method [27]. Amplicon pooling, sequencing on an Illumina HiSeq 2500 instrument using a modified PE300 protocol, sequence data pre-processing, and taxonomic assignments were conducted as described by Holm et al. [28]. Vaginal microbiota were assigned community state types (CSTs) [29] using VALENCIA (https://github.com/ravel-lab/VALENCIA), a nearest-centroid-based algorithm [30]. Four CSTs indicated dominance by Lactobacillus—*L. crispatus* (CST I), *L. gasseri* (CST II), *L. iners* (CST III), or *L. jensenii* (CST V). CST IV, a low-*Lactobacillus* state, was divided based on the presence and relative abundance of anaerobic organisms—BVAB1 and *Gardnerella vaginalis* (CST IV-A), *G. vaginalis* and *Atopobium vaginae* (CST IV-B), *Prevotella* spp. and others (CST IV-C) [30, 31].

### Statistical analyses

In order to compare baseline behavioral characteristics between matched cases and controls, asymptotic symmetry and marginal homogeneity tests were used for categorical variables, McNemar’s chi-square tests for binary variables, and paired *t*-tests or Wilcoxon signed-rank tests for continuous variables. For samples with vaginal cytokine measurements below the lower limit of detection, a number halfway between 0 and the lowest measured value for that particular cytokine for that particular plate was imputed [32]. Cytokines that were below the level of quantitation in > 90% of all samples (IL-2, IL-3, IL-5, IL-9, IL-10, IL-15, TNF-β) were excluded from the analysis. One analyte, IL-1RA, was above the upper limit of quantitation in 96% of samples and excluded from the analysis. In seven samples one analyte other than IL-1RA was above the upper limit. These seven values were imputed as 10,000 pg/mL, the upper limit of quantitation for the assay. Wilcoxon signed-rank tests were used to assess baseline differences in vaginal cytokine levels between matched cases and controls and differences in cytokines between pre- and post-exposure samples. We assessed differences in pre-exposure sample CSTs between cases and controls with the marginal homogeneity test. The Shannon Diversity Index (SDI) was calculated to measure the diversity of bacterial species within each vaginal sample [33]. The pre-exposure sample SDI was subtracted from the post-exposure value; a positive change in SDI indicated greater bacterial species diversity in the post-exposure sample. We then compared the median SDI change values between cases and controls using the Wilcoxon signed-rank test. In addition, we assessed the Yue Clayton theta distance [34], which measured the similarity in bacterial communities between pre- and post-exposure samples, and compared the theta distances for cases and controls using the Wilcoxon signed-rank test.

Linear mixed-effects and generalized linear mixed-effects models were used to assess differences in individual cytokines before and after exposure, and by case–control status. In the linear mixed-effects models for the cytokine analysis, the outcome was the log_10_-transformed ratio of the post-exposure cytokine level and the pre-exposure cytokine level (i.e., the relative post-to-pre change), and a normally distributed random effect term was used to adjust for matched pairs. For the vaginal microbiota analysis, to assess post-to-pre differences in the relative abundance of specific bacterial taxa, we employed similar modeling methods. Linear mixed-effects models were used to assess differences in the most abundant bacterial taxa before and after exposure by case–control status. To calculate the post-to-pre relative abundance ratio outcome for each bacterial taxa, we added a constant (1) to the relative abundances in all samples and divided the post-exposure relative abundance value by the pre-exposure value to obtain a ratio (Formula 1), which was then log_10_-transformed. A normally distributed random effect term was included in the model to adjust for matched pairs.

Formula 1:

$$Ratio=\frac{(``Post" sample\; relative\; abundance) + 1 }{(``Pre" sample\; relative\; abundance ) + 1}$$

Principal component analysis (PCA) was also conducted to visualize the pre- to post-exposure cytokine ratio in cases versus controls. Following an approach presented by Arnold et al. [35], we defined a binary measurement for “inflammation” as having at least three out of seven predetermined inflammatory cytokines (IL-1α, IL-1β, IL-8, MIP-1α, MIP-1β, IP-10, and RANTES) in the upper quartile. MIP-1α and IP-10 were added to the list of inflammatory cytokines based on relevant data from Masson et al. [36] showing a strong association with HIV seroconversion. A logistic mixed-effects model adjusting for matched pairs and within-subject correlations between pre- and post-exposure measures was applied with the binary inflammation score as the outcome. All analyses were conducted using STATA v14 (Stata Corp, College Station, TX) and R. PCA plots were constructed utilizing SIMCA 15 (Umetrics AB, Umea, Sweden).

## Results

Paired pre- and post-exposure samples from 22 lubricant users and 22 race-matched controls were analyzed for a total of 88 samples. 63.6% of participants were African American, and the mean age was 30 years (SD 6.8) (Table 1). There were no statistically significant differences between cases and controls in terms of age, douching, hormonal contraception (HC) use, or number of sexual partners, though cases were more likely to report lubricant use in the prior 6 months as compared to controls (p = 0.03) (Table 1, also see Additional file 2: Table S5 for data). Although cases reported more condomless vaginal sex compared to cases (average reporting of 3 days versus 7 days, respectively), the difference was not statistically significant (p = 0.25; Fig. 1A, B).

**Table 1:** Demographic and behavioral characteristics

	Overall N = 44	Controls n = 22	Lubricant users n = 22
Age, Mean (SD)	29.8 (6.8)	30.5 (7.0)	29.1 (6.8)
Race			
African American	28 (63.6)	14 (63.6)	14 (63.6)
White	14 (31.8)	7 (31.8)	7 (31.8)
Latina	2 (4.6)	1 (4.6)	1 (4.6)
Douching frequency last 60 days			
None	32 (72.7)	15 (68.2)	17 (77.3)
Monthly	3 (6.8)	3 (13.6)	0 (0.0)
Every now and then	3 (6.8)	1 (4.6)	2 (9.1)
No answer	6 (13.6)	3 (13.6)	3 (13.6)
Vaginal Lubricant use last 60 days (Y)*	11 (25.6)	2 (9.1)	9 (42.9)
Current HC use (Y)	11 (25.0)	5 (22.7)	6 (27.3)
Number sex partners last 60 days			
0	3 (6.8)	2 (9.1)	1 (4.6)
1	37 (84.1)	19 (86.4)	18 (81.8)
2	4 (9.1)	1 (4.6)	3 (13.6)
Pre-exposure sample pH*			
4.0–4.5	25 (58.1)	12 (54.6)	13 (61.9)
4.6–5.0	10 (23.3)	6 (27.3)	4 (19.1)
> 5.0	8 (18.6)	4 (18.2)	4 (19.1)
Pre-exposure sample Nugent category			
No BV	23 (52.3)	13 (59.1)	10 (45.5)
Intermediate	8 (18.2)	2 (9.1)	6 (27.7)
BV	13 (29.6)	7 (31.8)	6 (27.7)
Pre-exposure sample CST**			
CST-I, *L. crispatus*-dominated	8 (18.2)	6 (28.6)	2 (9.1)
CST-II, *L. gasseri*-dominated	5 (11.4)	2 (9.5)	3 (13.6)
CST-III, *L. iners*-dominated	14 (31.8)	5 (23.8)	9 (40.9)
CST-IVA, Low *Lactobacillus*	7 (15.9)	3 (14.3)	4 (18.2)
CST-IVB, Low *Lactobacillus*	6 (13.6)	3 (14.3)	3 (13.6)
CST-IVC, Low *Lactobacillus*	2 (4.5)	1 (4.8)	1 (4.5)
CST-V, *L. jensenii*-dominated	1 (2.3)	1 (4.8)	0 (0.0)
Pre-exposure sample CST**			
CST-I/II/V, *L. crispatus/gasseri/jensenii*-dominated	14 (32.6)	9 (42.9)	5 (22.7)
CST-III, *L. iners*-dominated	14 (32.6)	5 (23.8)	9 (40.9)
CST-IVA/B/C, Low *Lactobacillus*	15 (34.9)	7 (33.3)	8 (36.4)

*HC*: hormonal contraception, *CST*: community state type. Cases were more likely to report vaginal lubricant use in the last 60 days (p = 0.03). There were no statistically significant differences between cases and controls in terms of age, race, douching, hormonal contraception (HC) use, number of sexual partners or pre-exposure pH, Nugent category, or CST (p > 0.05). *1 case missing data. **1 control missing data

### Cytokine analysis

Prior to exposure, the median eotaxin, Flt-3L, and PDGFab were significantly higher in cases as compared to controls (Additional file 1: Table S1) with a similar trend in VEGF. Among controls, Wilcoxon signed-rank tests indicated that when comparing cytokines pre- to post-exposure as continuous measures, the median MCP-3 and sCD40L levels were significantly higher before exposure (Additional file 1: Table S2), with a similar trend in IL1-7α. There were no significant differences when comparing median cytokines post- to pre-exposure within cases (lubricant users), though there was a trend towards higher median eotaxin pre-exposure (p = 0.05) (Additional file 1: Table S3).

**Fig. 2:**
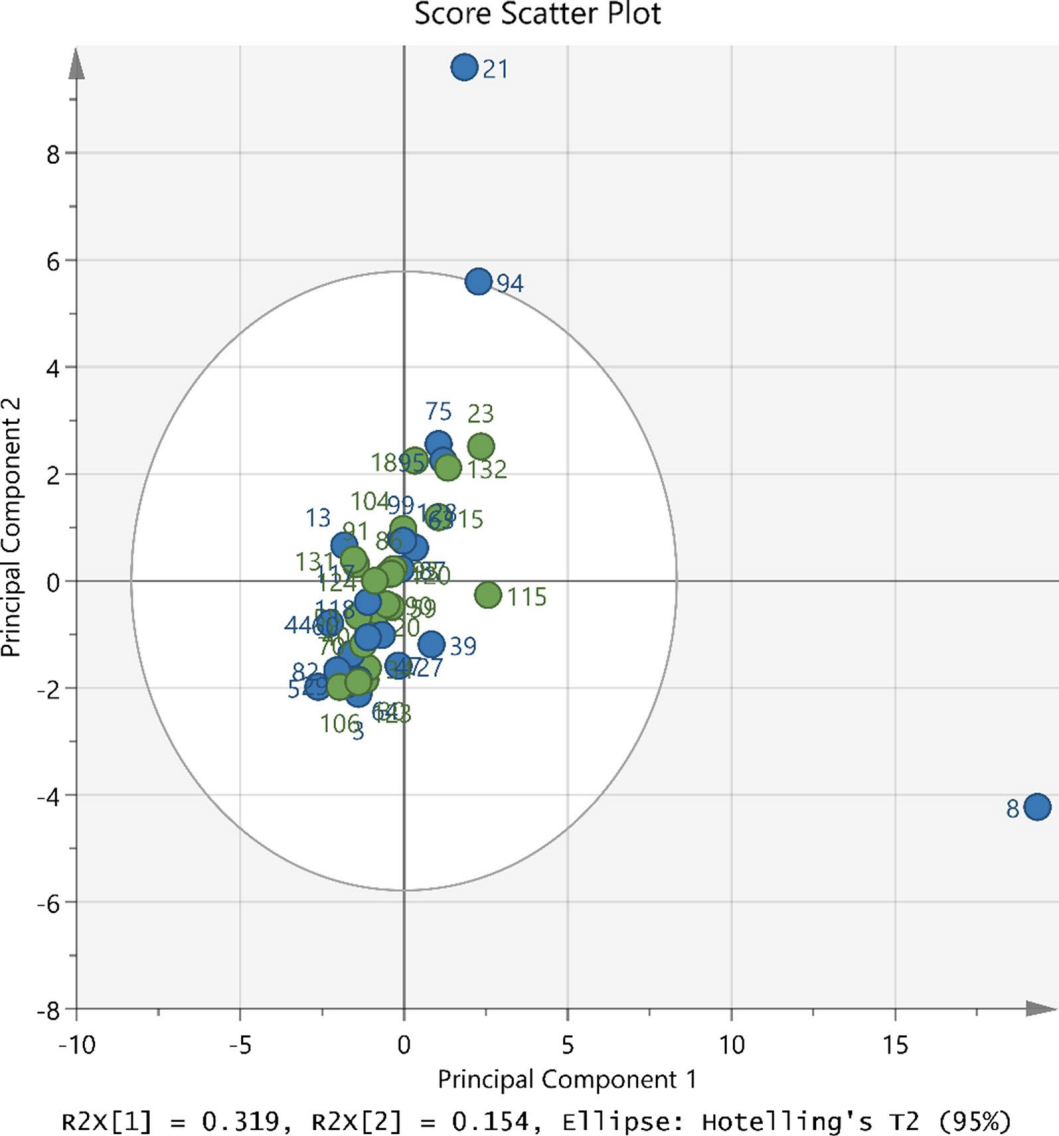
Principal components analysis of pre- to post-exposure cytokine ratios. Blue circles indicate cases (lubricant users) and green circles indicate controls

In mixed-effects modeling, adjusting for log-transformed baseline cytokine level, the mean log-transformed relative pre- to post-exposure change of cytokines was higher in cases as compared to controls for MDC (p = 0.03), (Additional file 1: Table S4). A PCA plot of the pre- to post-exposure cytokine ratios (Fig. 2) showed no clear grouping using the first two principal components by case–control status. Sample 8 had a higher value in the first principal component, while sample 21 had a higher value in the second principal component. These differences do not appear to be driven by any single cytokine.

Using a logistic mixed-effects model to analyze the binary inflammation score, there were no statistically significant differences in the odds of having an inflammatory vaginal microenvironment between cases and controls adjusting for whether it was sampled prior to or following exposure, within-subject correlations, and matched pairs (p = 0.16). Furthermore, there were no significant differences in the odds of having an inflammatory vaginal microenvironment between cases and controls in the pre-exposure samples after adjusting for matched pairs (p = 0.16). Finally, there were no significant differences in the odds of having an inflammatory vaginal microenvironment between pre- and post-exposure measures within controls (p = 0.14) or cases (p > 0.99) after adjusting for within-subject correlations. Due to the small sample size and exploratory intent of the pilot study, correction for multiple comparisons was not conducted, but none of our findings would have been significant after correction according to the Bonferroni method.

### Vaginal microbiota analysis

**Fig. 3:**
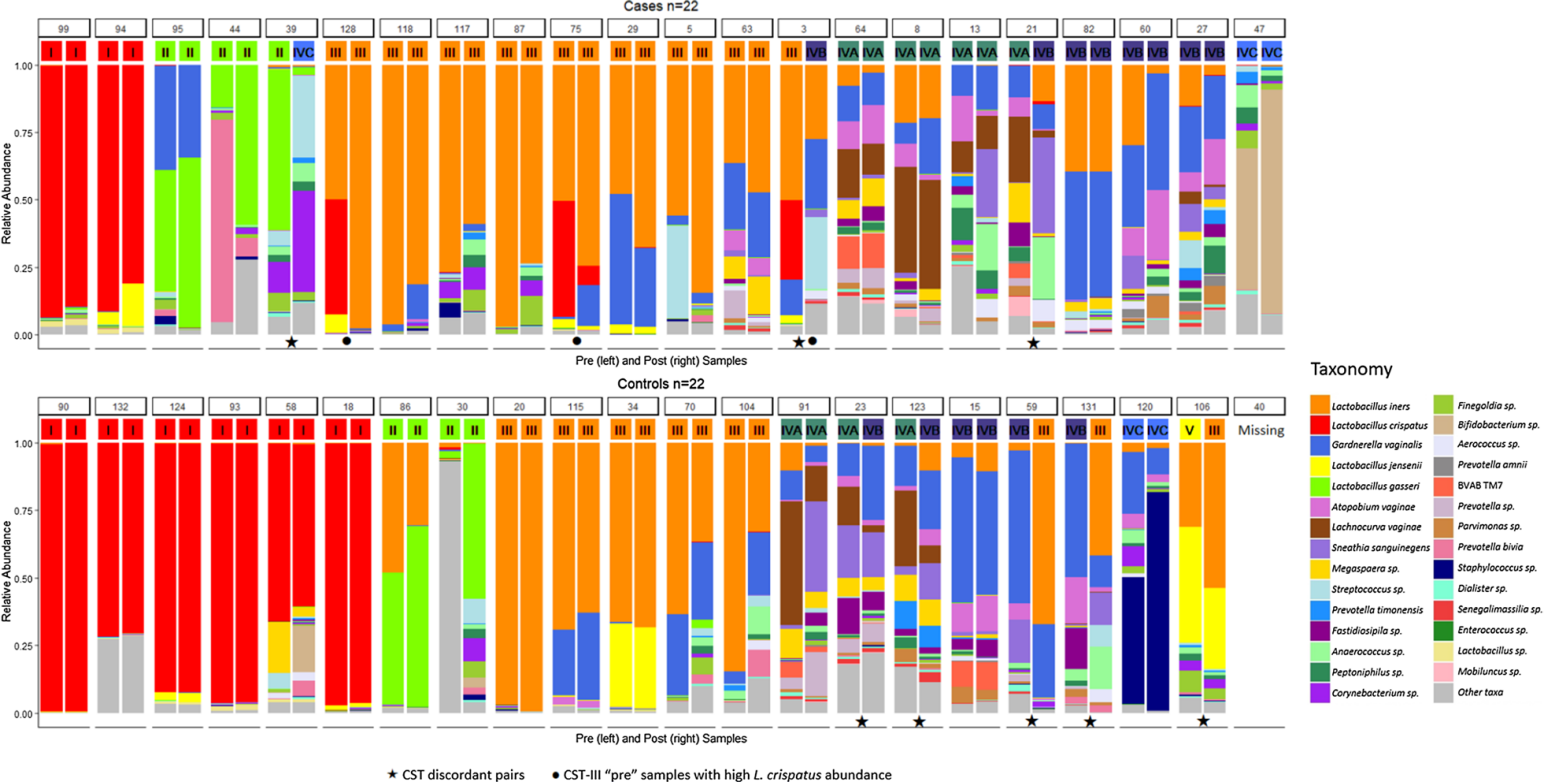
Bacterial relative abundances

The distribution of pre-exposure CSTs between cases and controls was similar whether a 7-CST (p = 0.56) or a 3-CST scheme (p = 0.29) were used, with the proportion of participants having a low-*Lactobacillus* sample prior to exposure being nearly equal in both groups (Table 1). The CSTs and bacterial taxa relative abundances of the samples included in the analysis are shown in Fig. 3. There were 3 (13%) CST-discordant case pairs (pre- and post-exposure samples) and 5 (24%) CST-discordant control pairs. The pre-to-post CST transitions observed were *Lactobacillus*-dominated to CST IV-B or CST IV-C (2 cases), CST IV-B to CST III (2 controls), CST IV-A to CST IV-B (1 case and 2 controls), and CST V to CST III (1 control).

The median change in SDI was -0.03 and 0.02 for cases and controls, respectively (Additional file 1: Figure S1A). These results suggest slightly higher species diversity in pre-exposure samples for cases and post-exposure samples for controls; however, the differences by case–control status were not statistically significant (p = 0.24). The median Yue Clayton theta distance was 0.72 for cases and 0.65 for controls indicating both had relatively high similarity, and there were no statistically significant differences in post-to-pre bacterial taxa between cases and controls (p = 0.52, Additional file 1: Figure S1B).

When focusing on the seven taxa with the highest relative abundance, there were no statistically significant differences between pre-exposure samples comparing cases and controls. The adjusted linear mixed-effects models showed a significant difference in the mean log_10_-transformed post-to-pre relative abundance between cases and controls only for *L. crispatus* (p = 0.014) (Table 2). When controlling for the pre-exposure sample *L. crispatus* relative abundance, the mean post-to-pre *L. crispatus* relative abundance ratio was lower (i.e., mean post-exposure relative abundance was lower than the pre-exposure relative abundance, producing a ratio < 1) in cases compared to controls. This finding is demonstrated in the relative abundance bar charts (Fig. 3). Among cases whose pre-exposure sample was classified as *L. iners*-dominated CST III, but also had a sizeable proportion of *L. crispatus* present, *L. crispatus* abundance was significantly lower after lubricant exposure in the post-exposure sample. There were no examples of controls with pre-exposure samples that were CST III with high *L. crispatus* relative abundance, and the same trend was not seen in cases or controls with pre-exposure samples that were classified as *L. crispatus*-dominated CST I.

**Table 2:** Top 7 most abundant taxa in the vaginal microbiota comparing mean post-to-pre-exposure ratio by case or control status

Taxa	Controls Mean post/pre ratio (SD)	Cases Mean post/pre ratio (SD)	Generalized mixed effect model p-value
*L. iners*	1.03 (0.19)	1.01 (0.12)	0.91
*L. crispatus*	1.00 (0.01)	0.96 (0.10)	0.01
*Gardnerella vaginalis*	1.00 (0.09)	1.02 (0.06)	0.10
*L. jensenii*	1.00 (0.02)	1.00 (0.03)	0.97
*L. gasseri*	1.03 (0.12)	1.01 (0.12)	0.46
*Atopobium vaginae*	0.99 (0.03)	1.00 (0.05)	0.40
*Candidatus Lachnocurva vaginae* (formerly BVAB1)	0.98 (0.06)	0.99 (0.04)	0.12

*SD*  standard deviation, *BVAB1*  bacterial vaginosis associated bacteria 1

## Discussion

Despite our hypothesis that we would observe a larger increase in pro-inflammatory cytokines and chemokines after lubricant use in this observational cohort, there were few statistically significant differences in vaginal cytokine profiles before and after exposure in lubricant users versus controls. For the three cytokines (Flt-3L, PDGFab, and eotaxin) which were statistically significantly different between the two groups at baseline, there was no statistically significant pre- to post-exposure change between groups in multivariate modeling. The post- to pre-exposure change of only one chemokine (MDC) was statistically significantly different between groups in multivariate modeling. MDC is a chemokine that recognizes the CCR4 receptor, is selective for Th2 cells, and expression has been shown to be induced by IL-4 and IL-13 [37–39]. MDC is one of seven cytokines that was found in a LASSO cytokine model to classify women as STI/BV positive or negative [21]. However, the lack of differences in MDC comparing pre- and post-exposure samples in lubricant users and the lack of differential change in other inflammatory cytokines or chemokines in lubricant users versus controls makes it difficult to interpret this isolated result. Certainly, in this cohort, there is no clear pattern to suggest a markedly pro-inflammatory vaginal microenvironment induced by lubricant use. Some previous studies (primarily from in vitro models) had raised concerns regarding the ability of lubricants (especially hyperosmolar lubricants) to lead to mucosal irritation and epithelial cell damage [6, 7, 9, 17, 40]. If true, this presumably would lead to a pro-inflammatory vaginal cytokine profile with potential implications for women’s reproductive health, including elevated risk of acquisition of STIs and HIV. While a single study found transcriptional upregulation of inflammatory genes in the upper reproductive tract with the use of a “universal placebo gel” previously thought to be inert [25], the results of our study are supported by results from two other placebo-controlled microbicide studies in which neither placebo vaginal lubricant nor microbicide resulted in increases in cervicovaginal pro-inflammatory cytokines [23, 24].

Other than direct cellular toxicity, concerns have been raised that lubricants could negatively impact the vaginal microbiota, leading to decreases in beneficial lactobacilli [9, 41], which in turn might have implications for reproductive health including increased susceptibility to STIs and HIV [31, 42]. We similarly hypothesized there would be a larger increase in strict and facultative anaerobic bacteria post-exposure in lubricant users compared to controls. We did not detect such a difference. Overall, we found few statistically significant differences at baseline between lubricant users or controls or changes in the overall structure of the vaginal microbiota before and after lubricant use. However, one taxon, *L. crispatus*, had higher relative abundance in pre-lubricant samples compared to post-exposure samples for cases. This is interesting given other studies have found a lack of *L. crispatus* dominance to be associated with a significant increase in susceptibility to HIV acquisition [13]. This is also noteworthy in light of several recent in vitro studies. The first found that the feminine moisturizer Vagisil inhibited *L. crispatus* growth [9]. Another found that two lubricant products (Replens Long-Lasting moisturizer and Trimo-San) inhibited the growth of *L. crispatus*, while another product (Replens Silky Smooth) enhanced growth [41]. A third paper found that lubricants containing chlorhexidine gluconate or nonoxynol-9 significantly inhibited *Lactobacillus* spp. growth, while other lubricants decreased the attachment of lactobacilli to vaginal epithelial cells in vitro [11]. These results, and ours, point to the need for larger, controlled studies to understand the impact of specific lubricants on the vaginal microbiome.

Animal and in vitro studies have suggested that exposure to semen may elicit the expression of pro-inflammatory cytokines in the vaginal tract [43, 44]. One study by Sharkey et al*.* demonstrated upregulation of several cytokine mRNAs in the human cervix, including IL-1α, IL-6, and IL-8, 12 h after condomless vaginal intercourse compared to 36 h prior [45]. We did not observe these changes in our analysis of pre- and post-vaginal sex exposures. In fact, of the cytokines or chemokines that were statistically significantly different in pre- and post-exposure samples (MCP-3 and sCD40L), all were lower post-vaginal sex than pre-vaginal sex. There could be several reasons for this. First, the area sampled and the analyses were different: the Sharkey study utilized cervical biopsies and looked for mRNA expression; we utilized vaginal swabs and directly measured secreted cytokine concentrations. Secondly, there was more variability in the timing of when our “post” sample was taken in relation to sexual intercourse (generally 12–36 h after). Furthermore, in the Sharkey study, patients used condoms for 5 days and then completely abstained from sexual intercourse for 2 days before the study was conducted. Our samples were taken from an observational cohort, and we did not require women to abstain from intercourse. It is possible that differences pre- and post-exposure to semen might be blunted in women engaging in regular sexual intercourse.

A strength of our study is the race-matched pre- to post-exposure design that enabled us to systematically assess real-world vaginal lubricant use in women compared to vaginal intercourse without lubricant, allowing us to assess if any findings were the result of lubricant use versus inflammatory changes following sexual activity/vaginal intercourse. We matched on race as it was strongly associated with lubricant use and CST in the Parent study, and based on prior literature, has been associated with several unmeasured factors (e.g., sexual networks and health behaviors) that may confound this analysis [46, 47]. We were unable to match on pre-exposure CST due to low sample size. However, future studies may investigate how various vaginal bacterial communities are affected by lubricant exposure, as CSTs may have varying resilience to extrinsic exposures.

Our research group [48, 49] and others [50–53] have validated self-collection of mid-vaginal swabs used for immunologic and microbiota analyses, and the practice is now widely used. The daily at-home sampling allows population-based longitudinal field studies. Our samples underwent two freeze thaw cycles. Several studies have shown low numbers (≤ 2) of freeze thaw cycles have negligible impact on the concentration of most cytokines [54–58]. Additionally, we focused on higher abundant bacterial taxa in this analysis. Data from gut microbiota literature suggests that storage of samples over hours to days at room temperature gives similar results to freezing immediately at –80°C, with only very low abundant taxa being affected [59–62]. Given the fact that samples were quickly frozen, we do not anticipate that the storage protocols would have affected the relative abundance of the relevant, highly abundant taxa.

However, there are significant limitations to our study. The daily diary did not collect information on which specific lubricants women used, and therefore, we were unable to assess whether these lubricants had elevated osmolalities or contained anti-microbial components. While there is a range of osmolalities and ingredients across commercially available personal lubricant products, in the period of time that this study was conducted, the osmolalities of commercially available lubricants were generally high [6]. Nonetheless, it is still possible in that women using frequent or large amounts of product, lubricants with elevated osmolalities or lubricants with anti-microbial components might have a different immune response. Secondly, since this was an exploratory analysis with a small sample size, we did not correct for multiple comparisons. None of our findings would have been statistically significant after correction for multiple comparisons. Thirdly, our method of imputation for undetectable cytokines, while widely used in the literature [32, 63], does have the potential to over or under-estimate true cytokine levels.

Additionally, we analyzed samples taken from an observational cohort study [26] that was powered for different aims. Small sample size was a limitation. The 22 cases and 22 controls represent a convenience sample from the original cohort, and we were only able to match one control per case on race. Furthermore, we did not mandate that women abstain from either condomless sexual intercourse or vaginal lubricant use prior to sample collection as it was an observational study. At baseline, women reported their vaginal lubricant use in the 60 days prior to enrollment in the study. Nine cases (41%) and two controls (9%) reported vaginal lubricant use in that period, including KY-Jelly (n = 7), Vaseline (n = 1), Silk (n = 1), mineral oil (n = 1), and an unspecified edible lubricant (n = 1). Although we confirmed that controls did not use any vaginal lubricants after enrollment, we were unable to verify whether cases continued use of the same lubricant product on the exposure day of interest. Post- to pre-exposure changes in cytokines might be blunted in women regularly engaging in condomless sexual intercourse or regularly utilizing vaginal lubricants. Again, because this was an observational study based on secondary data, the timing of sampling after intercourse/lubricant use was not rigidly controlled. While most post-exposure samples were taken between 12 and 36 h after intercourse, in 2 participants, this was substantially longer. It is possible that impacts on vaginal cytokines or microbiota are acute and short-lived (i.e., occurring shortly after use and quickly resolving), which could still lead to increased vulnerability (e.g., to HIV acquisition) but would not have been captured by our sampling methods. In addition, the methods used in this study to describe the vaginal microbiota do not differentiate between dead and live bacteria, and it is unknown how long DNA from dead bacteria can be detected in the vagina. It is possible that bacteria that were killed immediately after exposure would still be detectable in samples collected one day after exposure but not two or three days later. However, we posit that DNA detected from dead bacteria would constitute a small part of the result and not confound the observed associations.

Two participants (#44, a case, and #30, a control) had pre-exposure samples that had the highest similarity to the centroid of CST II by the VALENCIA algorithm. These two samples were dominated by *Klebsiella* and *P. bivia*, respectively but did contain a modest proportion of *L. gasseri* and were consequently placed in the *L. gasseri*-dominated CST II by the algorithm. In a sensitivity analysis, we reassigned these samples to CST IV-C; however, there was still no association between pre-exposure CST and lubricant use using marginal homogeneity tests. We also did not detect a difference in post-to-pre *L. gasseri* relative abundance between cases and controls.

Finally, we estimated the presence and relative abundance of key vaginal bacterial via 16S rRNA gene amplicon sequencing. However, it is possible that lubricant could affect not only the abundance of key bacteria but also their functional output—for example, the production of protective lactic acid from lactobacilli. We did not measure vaginal metabolites in this study. The observed difference in *L. crispatus* appears to be driven by cases whose pre-exposure samples were *L. iners*-dominated (CST III); however, there were no comparable instances among controls where a pre-exposure sample was *L. iners*-dominated but with a high relative abundance of *L. crispatus*, so it remains unknown what impact sex in the absence of lubricant use would have on this profile. Behavioral diaries were submitted weekly, and there may have been information bias in accurately reporting or recalling daily lubricant use or vaginal sex. We were not able to verify condomless vaginal sex or lubricant use with any biological measures.

## Conclusions

Although overall there were few differences in the vaginal microbiota and cytokine profiles of lubricant users and controls before and after condomless vaginal sex, there was a trend toward decreases in relative abundance of *L. crispatus* following use of lubricant. Within the limitations of the observational study design, our results are intriguing, but far from definitive. Larger, prospective, well-controlled studies with higher density sampling to carefully assess the impact of different types of over-the-counter vaginal lubricants, including those with high osmolarity, are needed to further evaluate the effect of vaginal lubricants on the vaginal microenvironment and inflammation.

## Supplementary Information

**Additional file 1: Table S1.** Comparing baseline vaginal cytokines prior to lubricant use with condomless vaginal sex (cases) to baseline cytokine profiles prior to condomless vaginal sex without lubricant use (controls): Wilcoxon signed-rank test. **Table S2.** Comparing vaginal cytokines before (“pre”) and after (“post”) condomless vaginal intercourse in N = 22 controls: Wilcoxon signed-rank test. **Table S3.** Comparing vaginal cytokines before (“pre”) and after (“post”) condomless vaginal intercourse with lubricant in N = 22 cases: Wilcoxon signed-rank test. **Table S4.** Multivariable modeling assessing differences in log-transformed pre-to-post ratio in cases versus controls. **Figure S1.** A. Change in Shannon diversity of the vaginal microbiota pre-to-post in cases compared to controls. B. Change in Yue Clayton theta distance of the vaginal microbiota pre-to-post in cases compared to controls.

**Additional file 2: Table S5.** Cytokine Concentrations and Metadata.

## Data Availability

The 16S rRNA gene sequences and CST assignments will be accessible using the National Center for Biotechnology Information (NCBI) Sequence Read Archive (SRA) BioProject accession number PRJNA208535, https://www.ncbi.nlm.nih.gov/bioproject/PRJNA208535. Cytokine concentration data and other demographic and health behavior metadata are included in Additional file 2: Table S5.
