# Supplementary material for: Vaginal cytokine profile and microbiota before and after lubricant use compared with condomless vaginal sex: a preliminary observational study
Source: BMC Infect Dis. 2021 Sep 18;21:973. doi: 10.1186/s12879-021-06512-x (PMC8449901; doi:10.1186/s12879-021-06512-x)

**Vaginal cytokine profile and microbiota before and after lubricant use compared with condomless vaginal sex: A preliminary observational study**

Susan Tuddenham^1*^, Christina A. Stennett^2*^, Richard A. Cone^3^, Jacques Ravel^2^, Andrew N. Macintyre^4^, Khalil G. Ghanem^1^, Xin He^5^, and Rebecca M. Brotman^2^

*Co-first authors: These two authors contributed equally to the manuscript.

1. Johns Hopkins University School of Medicine, Department of Medicine, Baltimore, MD, USA

2. University of Maryland School of Medicine, Institute for Genome Sciences, Baltimore, MD, USA

3. Johns Hopkins University, Baltimore, MD, USA

4. Duke University School of Medicine, Duke Human Vaccine Institute and Department of Medicine, Durham, NC, USA

5. University of Maryland College Park, School of Public Health, College Park, MD, USA

Corresponding Author:

Rebecca M. Brotman, PhD, MPH

Professor, Department of Epidemiology and Public Health

Institute for Genome Sciences

University of Maryland School of Medicine

670 West Baltimore Street, Room #3175

Baltimore, MD 21201

phone: (410) 706-6767

fax: (410) 706-1482

rbrotman@som.umaryland.edu

**Table S1. Comparing baseline vaginal cytokines prior to lubricant use with condomless vaginal sex (cases) to baseline cytokine profiles prior to condomless vaginal sex without lubricant use (controls): Wilcoxon signed rank test**

| Cytokine | Controls (N=22)  Median (Q1-Q3) | Cases (N=22)  Median (Q1-Q3) | P value |
| --- | --- | --- | --- |
| INFg | 1.6 (1.6-1.6) | 1.6 (1.6-1.6) | 0.47 |
| IL-1a | 122.7 (72.0-298.0) | 179.0 (87.3-282.9) | 0.61 |
| IL-1b | 2.6 (1.6-43.2) | 3.9 (1.6-21.1) | 0.58 |
| PDGFa | 1.6 (1.6-5.4) | 1.6 (1.6-9.6) | 0.45 |
| GCSF | 37.3 (9.9-57.8) | 50.9 (23-96.6) | 0.10 |
| Eotaxin | **5.2 (1.6-8.4)** | **7.6 (5.3-9.9)** | **0.02** |
| TGF-alpha | 5.6 (1.6-7.2) | 8.2 (5.3-23.9) | 0.14 |
| IL-6 | 1.6 (1.5-1.6) | 1.6 (1.5-7.3) | 0.38 |
| IL-8 | 330.9 (137.5-1674.1) | 542.4 (268.9-1569.1) | 0.57 |
| IL-12p40 | 6.6 (1.9-14.3) | 6.9 (5.9-15.1) | 0.13 |
| IL-12p70 | 1.7 (1.6-4.5) | 1.6 (1.6-4.1) | 0.98 |
| IL-17a | 1.6 (1.6-1.6) | 1.6 (1.6-3.4) | 0.28 |
| TNF-alpha | 1.6 (1.6-1.6) | 1.6 (1.6-1.6) | 0.98 |
| IL-4 | 10.2 (1.5-21.1) | 10.2 (8.4-18.7) | 0.54 |
| EGF | 28.6 (11.5-78.5) | 42.3 (24.7-58.4) | 0.78 |
| FGF | 38.9 (37.6-40.5) | 38.9 (37.6-40.5) | 0.63 |
| GM-CSF | 1.6 (1.6-3.5) | 1.6 (1.6-4.5) | 0.61 |
| INFa | 11.4 (1.5-24.9) | 17.2 (8.7-35.3) | 0.52 |
| IL-13 | 1.6 (1.6-1.6) | 1.6 (1.6-3.5) | 0.60 |
| IL-7 | 1.5 (1.5-4.7) | 4.5 (1.5-8.4) | 0.13 |
| MCP-1 | 56.3 (18.3-139.8) | 42.7 (9.1-141.4) | 0.73 |
| IP-10 | 85.8 (23.7-281.6) | 82.6 (37.6-186.5) | 0.45 |
| MIP-1a | 5.0 (1.7-7.6) | 5.5 (1.8-12.4) | 0.80 |
| MIP-1b | 5.6 (1.5-16.1) | 7.2 (2.7-18.6) | 0.22 |
| VEGF | 37.9 (25.6-47.1) | 47.6 (35.9-73.0) | 0.05 |
| Flt-3L | **7.3 (7.1-8.7)** | **14.3 (7.3-19.4)** | **0.04** |
| Fractalkine | 47.6 (38.6-50.3) | 50.3 (46.9-66.1) | 0.32 |
| GRO | 575.6 (181.0-1047.4) | 802.6 (266.5-2188.5) | 0.31 |
| MCP-3 | 10.5 (7.1-19.8) | 7.4 (6.1-17.9) | 0.96 |
| MDC | 63.4 (41.5-110.7) | 90.0 (58.6-138.5) | 0.18 |
| PDGFab | **16.0 (8.2-31.2)** | **25.2 (11.7-39.9)** | **0.04** |
| sCD40L | 1.6 (1.5-4.3) | 1.6 (1.5-3.4) | 0.71 |
| CCL5 | 3.7 (1.6-10.9) | 6.0 (3.1-10.4) | 0.56 |

Figure legend for abbreviations: interferon gamma (INFg), interleukin 1 alpha (IL-1a), interleukin 1 beta (IL-1b), interleukin 6 (IL-6), interleukin 8 (IL-8), interleukin 12 heterodimer p40 and p35 (IL12p70), interleukin 12 homodimer p40 (IL12p40), interleukin 17 alpha (IL-17a), tumor necrosis factor alpha (TNF-alpha), interleukin 4 (IL4), epidermal growth factor (EGF), fibroblast growth factor (FGF), granulocyte colony stimulating factor (GCSF), granulocyte-macrophage colony-stimulating factor (GM-CSF), interferon alpha (INFa), interleukin 13 (IL-13), interleukin IL-7, monocyte chemotactic protein 1 (MCP-1), interferon gamma-induced protein 10 (IP-10), macrophage Inflammatory protein 1 alpha (MIP-1a), macrophage inflammatory protein 1 beta (MIP-1b), vascular endothelial growth factor (VEGF), fms-like tyrosine kinase 3 ligand (Flt-3L), fractalkine, growth related protein (GRO), monocyte chemotactic protein-3 (MCP-3), macrophage derived chemokine (MDC), platelet derived growth factor AA dimer (PDGFa), platelet derived growth factor AB and BBdimer (PDGFab), soluble CD40 ligand (sCD40L), RANTES (CCL5), eotaxin, and transforming growth factor alpha (TGF-alpha).

**Table S2. Comparing vaginal cytokines before (“pre”) and after (“post”) condomless vaginal intercourse in N=22 controls: Wilcoxon signed rank test**

| Cytokine | Pre  Median (Q1-Q3) | Post  Median (Q1-Q3) | P value |
| --- | --- | --- | --- |
| INFg | 1.6 (1.6-1.6) | 1.6 (1.6-1.6) | 0.26 |
| IL-1a | 122.7 (72.0-298.0) | 128.0 (54.6-267.6) | 0.17 |
| IL-1b | 2.6 (1.6-43.2) | 1.6 (1.6-18.2) | 0.93 |
| PDGFa | 1.6 (1.6-5.4) 25.5 (89.1) | 4.2 (1.6-12.1)) | 0.25 |
| GCSF | 37.3 (9.9-57.8) | 38.5 (9.9-57.6) | 0.57 |
| Eotaxin | 5.2 (1.6-8.4) | 5.0 (3.5-7.1) | 0.81 |
| TGF-alpha | 5.6 (1.6-7.2) | 5.8 (1.6-10.4) | 0.60 |
| IL-6 | 1.6 (1.5-1.6) | 1.6 (1.5-1.6) | 0.69 |
| IL-8 | 330.9 (137.5-1674.1) | 481.2 (265.3-721.6) | 0.41 |
| IL-12p40 | 6.6 (1.9-14.3) | 6.6 (1.9-11.3) | 0.43 |
| IL-12p70 | 1.7 (1.6-4.5) | 1.6 (1.6-1.7) | 0.20 |
| IL-17a | 1.6 (1.6-1.6) | 1.6 (1.6-1.6) | 0.05 |
| TNF-alpha | 1.6 (1.6-1.6) | 1.6 (1.6-1.6) | 0.28 |
| IL-4 | 10.2 (1.5-21.1) | 10.2 (1.5-25.9) | 0.95 |
| EGF | 28.6 (11.5-78.4) | 45.5 (16.0-101.2) | 0.39 |
| FGF | 38.9 (37.6-40.5) | 38.8 (37.6-40.5) | 0.09 |
| GM-CSF | 1.6 (1.6-3.5) | 1.6 (1.6-1.6) | 0.16 |
| INFa | 11.4 (1.5-24.9) | 20.2 (1.5-41.7) | 0.78 |
| IL-13 | 1.6 (1.6-1.6) | 1.6 (1.6-1.6) | 0.47 |
| IL-7 | 1.5 (1.5-4.7) | 1.5 (1.5-6.3) | 0.49 |
| MCP-1 | 56.3 (18.3-139.8) | 23.2 (5.6-143.8 | 0.07 |
| IP-10 | 85.8 (23.7-281.6) | 103.2 (39.6-220.8) | 0.39 |
| MIP-1a | 5.0 (1.7-7.6) | 5.4 (1.7-7.3) | 0.88 |
| MIP-1b | 5.6 (1.5-16.1) | 3.8 (1.5-11.1) | 0.28 |
| VEGF | 37.9 (25.6-47.1) | 44.2 (23.8-56.0) | 0.95 |
| Flt-3L | 7.3 (7.1-8.7) | 7.3 (7.1-14.4) | 0.51 |
| Fractalkine | 47.6 (38.6-50.3) | 49.0 (33.3-53.3) | 0.63 |
| GRO | 575.6 (181.0-1047.4) | 514.3 (51.6-2566.5) | 0.54 |
| MCP-3 | **10.5 (7.1-19.8)** | **7.1 (6.1-13.2)** | **0.04** |
| MDC | 63.4 (41.5-110.7) | 77.5 (21.4-134.1) | 0.96 |
| PDGFab | 16.0 (8.2-31.2) | 15.7 (10.3-31.7) | 0.90 |
| sCD40L | **1.6 (1.5-4.3)** | **1.6 (1.5-1.6)** | **0.03** |
| CCL5 | 3.7 (1.6-10.9) | 3.6 (1.6-5.7) | 0.62 |

Figure legend for abbreviations: interferon gamma (INFg), interleukin 1 alpha (IL-1a), interleukin 1 beta (IL-1b), interleukin 6 (IL-6), interleukin 8 (IL-8), interleukin 12 heterodimer p40 and p35 (IL12p70), interleukin 12 homodimer p40 (IL12p40), interleukin 17 alpha (IL-17a), tumor necrosis factor alpha (TNF-alpha), interleukin 4 (IL4), epidermal growth factor (EGF), fibroblast growth factor (FGF), granulocyte colony stimulating factor (GCSF), granulocyte-macrophage colony-stimulating factor (GM-CSF), interferon alpha (INFa), interleukin 13 (IL-13), interleukin IL-7, monocyte chemotactic protein 1 (MCP-1), interferon gamma-induced protein 10 (IP-10), macrophage Inflammatory protein 1 alpha (MIP-1a), macrophage inflammatory protein 1 beta (MIP-1b), vascular endothelial growth factor (VEGF), fms-related tyrosine kinase 3 ligand (Flt-3L), fractalkine, growth related protein (GRO), monocyte chemotactic protein-3 (MCP-3), macrophage derived chemokine (MDC), platelet derived growth factor AA dimer (PDGFa), platelet derived growth factor AB and BBdimer (PDGFab), soluble CD40 ligand (sCD40), RANTES (CCL5), eotaxin, and transforming growth factor alpha (TGF-alpha).

**Table S3. Comparing vaginal cytokines before (“pre”) and after (“post”) condomless vaginal intercourse with lubricant in N=22 cases: Wilcoxon signed rank test**

| Cytokine | Pre  Median (Q1-Q3) | Post  Median (Q1-Q3) | P value |
| --- | --- | --- | --- |
| INFg | 1.6 (1.6-1.6) | 1.6 (1.6-3.8) | 0.66 |
| IL-1a | 179.0 (87.3-283.0) | 199.7 (103.8-574.5) | 0.08 |
| IL-1b | 3.9 (1.6-21.1) | 4.3 (1.6-47.2) | 0.61 |
| PDGFa | 1.6 (1.6-9.6) | 7.0 (1.6-17.2) | 0.16 |
| GCSF | 50.9 (23.0-96.6) | 67.6 (15.9-257.9) | 0.48 |
| Eotaxin | 7.6 (5.3-9.9) | 6.8 (3.9-8.1) | 0.05 |
| TGF-alpha | 8.2 (5.3-23.9) | 7.8 (5.2-11.4) | 0.27 |
| IL-6 | 1.6 (1.5-7.3) | 1.6 (1.5-4.8) | 0.79 |
| IL-8 | 542.4 (268.9-1569.1) | 639.5 (226.3-1519.9) | 0.68 |
| IL-12p40 | 6.9 (5.9-15.1) | 9.6 (6.6-14.5) | 0.94 |
| IL-12p70 | 1.6 (1.6-4.1) | 1.6 (1.6-3.5) | 0.82 |
| IL-17a | 1.6 (1.6-3.4) | 1.6 (1.6-1.6) | 0.32 |
| TNF-alpha | 1.6 (1.6-1.6) | 1.6 (1.6-1.6) | 0.17 |
| IL-4 | 10.2 (8.4-18.7) | 10.1 (4.2-15.8) | 0.21 |
| EGF | 42.3 (24.7-58.4) | 63.4 (22.8-82.5) | 0.34 |
| FGF | 38.9 (37.6-40.5) | 38.9 (37.6-40.5) | 0.26 |
| GM-CSF | 1.6 (1.6-4.5) | 1.6(1.6-1.6) | 0.64 |
| INFa | 17.2 (8.7-35.3) | 10.3 (7.5-27.8) | 0.18 |
| IL-13 | 1.6 (1.6-3.5) | 1.6 (1.6-3.1) | 0.46 |
| IL-7 | 4.5 (1.5-8.4) | 4.4 (1.5-7.2) | 0.82 |
| MCP-1 | 42.7 (9.1-141.4) | 23.8 (12.2-100.9) | 0.27 |
| IP-10 | 82.6 (37.6-186.5) | 82.2 (30.8-261.9) | 0.59 |
| MIP-1a | 5.5 (1.8-12.4) | 6.5 (1.8-12.3) | 0.57 |
| MIP-1b | 7.2 (2.7-18.6) | 8.1 (3.0-13.2) | 0.17 |
| VEGF | 47.6 (35.9-73.0) | 45.7 (19.3-70.1) | 0.29 |
| Flt-3L | 14.3 (7.3-19.4) | 7.3 (7.1-15.5) | 0.10 |
| Fractalkine | 50.3 (46.9-66.1) | 49.6 (20.0-50.3) | 0.08 |
| GRO | 802.6 (266.5-2188.5) | 1115.7 (243.0-3080.9) | 0.37 |
| MCP-3 | 7.4 (6.1-17.9) | 7.4 (7.1-19.8) | 0.88 |
| MDC | 90.0 (58.6-138.5) | 104.2 (63.4-187.4) | 0.32 |
| PDGFab | 25.2 (11.7-39.9) | 19.6 (12.1-44.5) | 0.39 |
| sCD40L | 1.6 (1.5-3.4) | 1.6 (1.5-3.4) | 0.94 |
| CCL5 | 6.0 (3.1-10.4) | 5.7 (1.6-11.3) | 0.79 |

Figure legend for abbreviations: interferon gamma (INFg), interleukin 1 alpha (IL-1a), interleukin 1 beta (IL-1b), interleukin 6 (IL-6), interleukin 8 (IL-8), interleukin 12 heterodimer p40 and p35 (IL12p70), interleukin 12 homodimer p40 (IL12p40), interleukin 17 alpha (IL-17a), tumor necrosis factor alpha (TNF-alpha), interleukin 4 (IL4), epidermal growth factor (EGF), fibroblast growth factor (FGF), granulocyte colony stimulating factor (GCSF), granulocyte-macrophage colony-stimulating factor (GM-CSF), interferon alpha (INFa), interleukin 13 (IL-13), interleukin IL-7, monocyte chemotactic protein 1 (MCP-1), interferon gamma-induced protein 10 (IP-10), macrophage Inflammatory protein 1 alpha (MIP-1a), macrophage inflammatory protein 1 beta (MIP-1b), vascular endothelial growth factor (VEGF), fms-like tyrosine kinase 3 ligand (Flt-3L), fractalkine, growth related protein (GRO), monocyte chemotactic protein-3 (MCP-3), macrophage derived chemokine (MDC), platelet derived growth factor AA dimer (PDGFa), platelet derived growth factor AB and BBdimer (PDGFab), soluble CD40 ligand (sCD40L), RANTES (CCL5), eotaxin, and transforming growth factor alpha (TGF-alpha).

**Table S4. Multivariable modeling assessing differences in log transformed pre-to-post ratio in cases versus controls.**

| Cytokine | Post/Pre Ratio Controls  Mean (SD)* | Post/Pre Ratio Cases  Mean (SD)* | Adjusted P value** |
| --- | --- | --- | --- |
| INFg | 1.0 (0.6) | 1.7 (2.9) | 0.22 |
| IL-1a | 1.5 (1.3) | 2.0 (1.8) | 0.25 |
| IL-1b | 2.4 (4.2) | 3.8 (7.6) | 0.24 |
| PDGFa | 5.5 (10.4) | 11.4 (30.5) | 0.59 |
| GCSF | 2.3 (2.9) | 2.2 (3.0) | 0.90 |
| Eotaxin | 1.3 (0.9) | 0.9 (0.6) | 0.61 |
| TGF-alpha | 1.5 (1.3) | 1.1 (0.8) | 0.66 |
| IL-6 | 1.1 (0.8) | 1.9 (2.7) | 0.24 |
| IL-8 | 1.6 (1.4) | 1.9 (2.2) | 0.36 |
| IL-12p40 | 2.0 (3.5) | 2.1 (3.7) | 0.25 |
| IL-12p70 | 0.9 (0.6) | 3.9 (13.2) | 0.27 |
| IL-17a | 0.9 (0.3) | 1.4 (2.1) | 0.11 |
| TNF-alpha | 1.0 (0.6) | 1.0 (0.8) | 0.28 |
| IL-4 | 1.5 (1.7) | 1.8 (3.0) | 0.94 |
| EGF | 2.2 (1.8) | 1.9 (2.4) | 0.63 |
| FGF | 1.1 (1.0) | 4.0 (13.2) | 0.91 |
| GM-CSF | 1.6 (3.5) | 2.4 (6.3) | 0.83 |
| INFa | 6.8 (24.1) | 1.9 (4.2) | 0.22 |
| IL-13 | 3.8 (12.2) | 1.1 (0.8) | 0.40 |
| IL-7 | 2.0 (3.5) | 1.2 (1.2) | 0.93 |
| MCP-1 | 1.2 (1.6) | 3.8 (9.9) | 0.22 |
| IP-10 | 1.9 (3.1) | 2.1 (3.8) | 0.77 |
| MIP-1a | 1.4 (1.3) | 1.5 (1.5) | 0.85 |
| MIP-1b | 1.0 (0.7) | 1.9 (3.7) | 0.45 |
| VEGF | 1.9 (4.2) | 1.0 (0.8) | 0.48 |
| Flt-3L | 1.2 (0.7) | 1.0 (0.8) | 0.49 |
| Fractalkine | 1.0 (0.4) | 1.6 (4.0) | 0.37 |
| GRO | 1.5 (1.8) | 3.5 (5.3) | 0.24 |
| MCP-3 | 0.8 (0.5) | 1.6 (1.6) | 0.24 |
| MDC | **1.3 (0.9)** | **1.9 (1.9)** | **0.03** |
| PDGFab | 3.9 (6.0) | 1.9 (2.9) | 0.87 |
| sCD40L | 0.9 (0.3) | 1.9 (3.6) | 0.64 |
| CCL5 | 6.1 (23.3) | 4.2 (9.3) | 0.61 |

Figure legend for abbreviations: interferon gamma (INFg), interleukin 1 alpha (IL-1a), interleukin 1 beta (IL-1b), interleukin 6 (IL-6), interleukin 8 (IL-8), interleukin 12 heterodimer p40 and p35 (IL12p70), interleukin 12 homodimer p40 (IL12p40), interleukin 17 alpha (IL-17a), tumor necrosis factor alpha (TNF-alpha), interleukin 4 (IL4), epidermal growth factor (EGF), fibroblast growth factor (FGF), granulocyte colony stimulating factor (GCSF), granulocyte-macrophage colony-stimulating factor (GM-CSF), interferon alpha (INFa), interleukin 13 (IL-13), interleukin IL-7, monocyte chemotactic protein 1 (MCP-1), interferon gamma-induced protein 10 (IP-10), macrophage Inflammatory protein 1 alpha (MIP-1a), macrophage inflammatory protein 1 beta (MIP-1b), vascular endothelial growth factor (VEGF), fms-like tyrosine kinase 3 ligand (Flt-3L), fractalkine, growth related protein (GRO), monocyte chemotactic protein-3 (MCP-3), macrophage derived chemokine (MDC), platelet derived growth factor AA dimer (PDGFa), platelet derived growth factor AB and BBdimer (PDGFab), soluble CD40 ligand (sCD40L), RANTES (CCL5), eotaxin, and transforming growth factor alpha (TGF-alpha). *Unadjusted **Based on adjusted comparisons

**Figure S1A. Change in Shannon Diversity of the Vaginal Microbiota Pre-to-Post in Cases compared to Controls.**

**
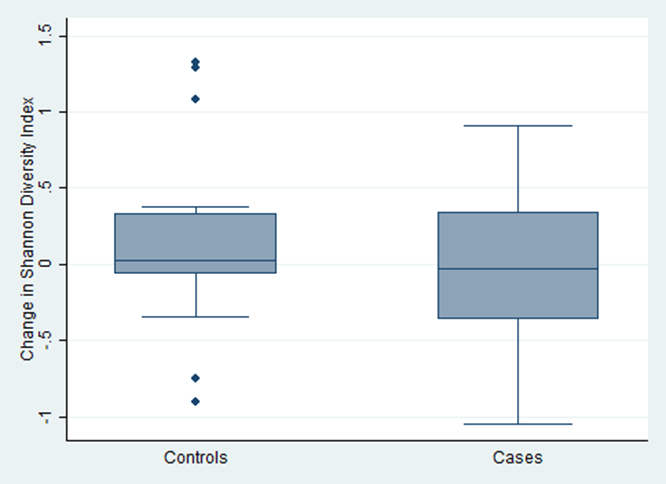
**

**Figure S1B. Change in Yue Clayton Theta Distance of the Vaginal Microbiota Pre-to-Post in Cases compared to Controls.**


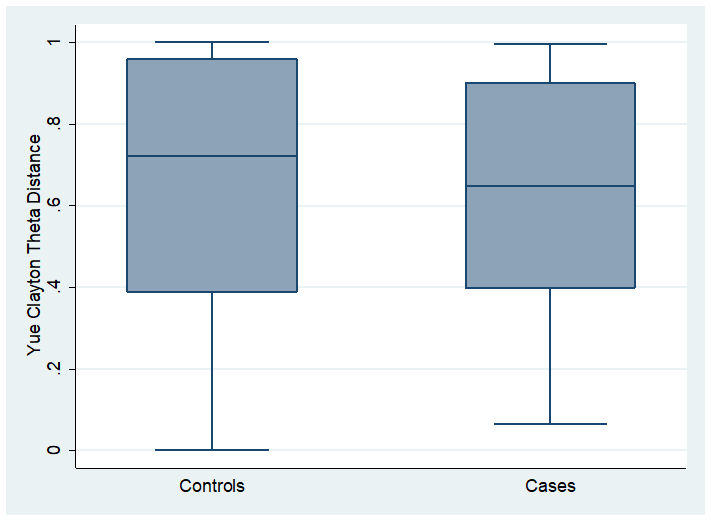

Supplement: Supplementary file 1 — Additional file 1: Table S1. Comparing baseline vaginal cytokines prior to lubricant use with condomless vaginal sex (cases) to baseline cytokine profiles prior to condomless vaginal sex without lubricant use (controls): Wilcoxon signed-rank test. Table S2. Comparing vaginal cytokines before (“pre”) and after (“post”) condomless vaginal intercourse in N = 22 controls: Wilcoxon signed-rank test. Table S3. Comparing vaginal cytokines before (“pre”) and after (“post”) condomless vaginal intercourse with lubricant in N = 22 cases: Wilcoxon signed-rank test. Table S4. Multivariable modeling assessing differences in log-transformed pre-to-post ratio in cases versus controls. Figure S1. A. Change in Shannon diversity of the vaginal microbiota pre-to-post in cases compared to controls. B. Change in Yue Clayton theta distance of the vaginal microbiota pre-to-post in cases compared to controls. [file 12879_2021_6512_MOESM1_ESM.docx]
